# Gene polymorphisms influencing yield, composition and technological properties of milk from Czech Simmental and Holstein cows

**DOI:** 10.5713/ajas.19.0520

**Published:** 2020-01-13

**Authors:** Jindřich Čítek, Michaela Brzáková, Lenka Hanusová, Oto Hanuš, Libor Večerek, Eva Samková, Zuzana Křížová, Irena Hoštičková, Tereza Kávová, Karolina Straková, Lucie Hasoňová

**Affiliations:** 1 Faculty of Agriculture, University South Bohemia, CZ37005 Ceske Budejovice, Czech Republic; 2 Dairy Research Institute, CZ16000 Prague, Czech Republic

**Keywords:** Dairy Cattle, Performance, Milk Fermentation Ability, Renneting, Polymorphisms

## Abstract

**Objective:**

The aim of the study was to evaluate the influence of polymorphic loci and other factors on milk performance and the technological properties of milk.

**Methods:**

The analysis was performed on Simmental and Holstein cows in field conditions (n = 748). Milk yield in kg, fat and protein percentage and yield were evaluated. Technological properties were evaluated by milk fermentation ability, renneting, and an alcohol test. Polymorphisms in the acyl-CoA diacylgycerol transferase 1 (*DGAT1*), leptin (*LEP*), fatty acid synthase (*FASN*), stearoyl CoA desaturase 1 (*SCD1*), casein beta (*CSN2*), casein kappa (*CSN3*), and lactoglobulin beta genes were genotyped, and association analysis was performed.

**Results:**

The *DGAT1* AA genotype was associated with higher milk, protein and fat yields (p<0.05). The MM genotype in the *LEP* gene was associated with a lower protein percentage and the W allele with a higher protein percentage (p<0.05). In cows with the *FASN* GG genotype, the protein percentage was higher, but the A allele was associated with higher milk, protein and fat yields than the G allele. The TT genotype in *SCD1* was associated with the lowest milk, protein and fat yields and with the highest milk protein percentage (p<0.01). The T allele had higher values than the C allele (p<0.05) except for fat percentage. The genotype *CSN3* AA was associated with a significantly heightened milk yield; BB was associated with a high protein percentage. The effect of the alleles on the technological properties was not significant. The *CSN2* BB genotype was associated with the best alcohol test (p<0.01), and the renneting order was inverse. Milk from cows with the *CSN2* A^1^A^1^ genotype was best in the milk fermentation ability. *CSN3* significantly affected the technological properties.

**Conclusion:**

The findings revealed the potential of some polymorphic loci for use in dairy cattle breeding and for the management of milk quality. In field research, the pivotal role of farms in milk yield, composition and technological properties was confirmed.

## INTRODUCTION

Milk yield and composition substantially impact the economics of dairy farms. For milk manufacturing, in addition to the percentages of fat and protein and the microbial quality, the technological properties of milk are important, as cheese has become a very important product in the dairy industry and enzymatic coagulation of milk is a crucial step in cheese-making process [1]. The influence of diet on the cheese-making properties of milk is often analyzed [2]. Other impacts on technological properties, such as the housing system or the stage of lactation, have also been the focus of studies [3,4].

Cecchinato et al [5] studied milk coagulation properties and curd firmness and found heritability of up to 0.278. Part of the heritability seems to be due to the polymorphisms in major genes analyzed in their work. They confirmed some previously documented associations, e.g., those between casein beta (*CSN2*) and technological properties, and identified a number of novel associations. They reported that different genes are involved in the coagulation phase. An interesting study of *CSN2*, casein kappa (*CSN3*), and lactoglobulin beta (*LGB*) genes was performed on Czech Simmental cows [6]. The authors confirmed that first-parturition cows show a certain shift and imbalance in milk physiochemical parameters, and the effect of the composite genotype on the investigated traits mostly reflected the effects of the individual genes. Genetic polymorphism has been related to milk titratable acidity, alcohol stability, phosphorus and calcium contents in milk, yogurt pH and the number of fermenting *Lactobacilli*. Their findings support the previously accepted indirect effects of milk protein polymorphisms on milk technological quality. The authors propose to perform a detailed analysis of polymorphisms and the interaction of the genes in larger populations to confirm their results and to find differences among breeds. They suppose that further study of the milk protein composite genotypes in cattle may have future implications for the production of milk with defined characteristics.

Recently, acyl-CoA diacylgycerol transferase 1 (*DGAT1*) polymorphisms were studied. The lysine variant positively influenced the breeding value for fat content in milk [7]. The polymorphism of *DGAT1* in Jersey, Holstein-Friesian and Ayrshire breeds in New Zealand influenced the yield of fat, protein and milk [8]. Other authors found significant association to total not fat solid, fat and protein contents, but not milk yield [9]. Stearoyl CoA desaturase 1 (*SCD1*) is the other gene studied, their polymorphisms were found to be associated with fat content [10]. Animals with the CC genotype compare favourably with individuals with other genotypes in terms of milk yield [11]. Also, *LEP*, fatty acid synthase (*FASN*) and other gene polymorphisms were studied [12,13].

The aim of this paper was to evaluate the association of polymorphisms in the *DGAT1*, *LEP*, *FASN*, *SCD1*, *CSN2*, *CSN3*, and *LGB* genes with the performance, composition and technological qualities of cow milk. Milk protein genes with the potential to affect milk quality were chosen, i.e. caseins and lactoglobulin beta. Moreover, *DGAT1*, *LEP*, *FASN*, and *SCD1* genes were involved. These genes are studied in regard to the milk performance, but the influence of their polymorphisms on technological qualities of milk was not evaluated adequately so far. Analysis was performed on numerous animals in field conditions in several farms, and additional factors affecting the performance and quality of milk were also evaluated.

## MATERIALS AND METHODS

### Animals

All experiments were performed in accordance with relevant guidelines and regulations recommended by the Ministry of Agriculture of the Czech Republic. All animal experiments were under supervision of the Institutional Animal Care and Use Committee of the Faculty of Agriculture of the South Bohemia University, where the experiment was carried out, approval number 22036/2019-MZE-18134. DNA was extracted noninvasively from milk samples.

The analysis was performed in cows of the Czech Simmental (part of the Simmental group) and Holstein breeds and their crosses; the cows were kept in the Czech Republic. As the crossbreds of Holstein and Simmental with small proportions of Ayrshire are common in herds in the Czech Republic, they were included into our field research as well. The numbers of purebred and crossbred cows are given in Supplementary Table S1. Cows were kept by five companies in free housing and fed with maize silage, grass silage, hay and feed concentrates year-round. The ratios differed among companies in terms of the share of constituents and their quality. The cows calved from 2015 through 2017. The 1st lactation was recorded for 748 cows, and the 2nd was also recorded for 660 of those cows. The mean milk yield was 8,036 kg in the 1st lactation and 8,722 kg in the 2nd lactation. The fat percentages were 4.12 and 4.12, the crude protein percentages were 3.46 and 3.48, the fat yields were 329.8 kg and 358.0 kg, and the crude protein yields were 274.8 kg and 301.3 kg for the first and second lactations, respectively.

The technological properties (milk fermentation ability, renneting measured by classical procedure and by nephelometry, ethanol test) of milk samples from 242 cows were examined. Of these cows, 81 were sampled once, 86 twice, 53 three times, 16 four times, and 6 five times. The cows were sampled throughout the course of the year.

### Genotyping

Milk samples were individually collected, and DNA was isolated using the DNA/RNA extractor MagCore HF16 Plus (RBC Bioscience, New Taipei, Taiwan). Isolation was performed according to the manufacturer’s instructions using the MagCore DNA Whole Blood Kit and MagCore Genomic DNA Tissue Kit (RBC Bioscience, Taiwan). The quality and quantity of the isolated DNA were verified by electrophoresis and spectrophotometry.

Genotyping of all loci was performed by the polymerase chain reaction and restriction fragment length polymorphism (PCR/RFLP) method. *DGAT1* gene alleles A (alanine) and K (lysine) were genotyped according to the methods of Kuhn et al [14]; *LEP* gene alleles M and W according to Buchanan et al [15]; *FASN* gene alleles A and G according to Roy et al [16]; *CSN2* gene alleles A and B according to Medrano and Sharrow [17]; and alleles A^1^ and A^2^ according to Miluchová et al [18]. *CSN3* gene alleles A, B, C, and E were analyzed according to the methodology of Barroso et al [19]; *LGB* alleles A and B according to Strzalkowska et al [20]; and *SCD1* gene alleles C and T according to Inostroza et al [21]. The primer sequences are given in Supplementary Table S2.

The resulting genotypes were electrophoretically determined, and genotype and allelic frequencies were calculated. To evaluate the Hardy-Weinberg Equilibrium, the differences between the observed and expected frequencies of the genotypes were tested using a χ^2^ test with the significance level p<0.05 and p<0.01. Supplementary Table S3 gives the frequencies of genotypes and alleles of all genotyped Simmental, Holstein and crossbred cows.

### Milk performance, composition and analysis of technological qualities

Data on milk performance were collected from the milk recording breeder’s database. Milk yield in kg, fat and crude protein percentage, and fat and crude protein yield in kg were evaluated. Milk composition (fat and crude protein contents) was determined in breeder milk laboratories of the Czech-Moravia Breeders Association using infrared spectroscopy (Foss Electric, Foss A/S, Hilleroed, Denmark; and Bentley Instruments, Chaska, MN, USA) instrumentation. These laboratories are accredited to the ISO standard (CSN EN ISO/IEC 17025) for official milk performance analysis in the Czech Republic and are working under the ICAR (International Committee for Animal Recording) umbrella (ICAR certificate to Czech Moravian Breeders’ Corp, Hradistko; Accredited Milk Laboratory Bustehrad, Czech Republic, for identification of dairy cattle, production recording in dairy cattle, genetic evaluation, milk laboratory operation, linear classification/scoring, and data processing), regularly taking part in relevant proficiency testing. Analytical instruments were regularly monthly calibrated according to the reference method results (extraction by the Röse–Gottlieb method for fat content [22] and distillation and titration according to the Kjeldahl method for crude protein content (total nitrogen content ×6.38) [23]). Technological properties were evaluated by a milk fermentation ability test, renneting was measured subjectively and instrumentally, and an ethanol test was performed.

The milk ethanol stability was determined by milk titration (5 mL) with 96% ethanol until the first precipitation flakes of milk protein were visible and is reported as ml of alcohol. This procedure was modified according to Horne [24].

The milk fermentation ability of the yogurt test was carried out according to the Czech milk industry standards. A sample of raw milk (50 mL) was heated at 85°C for 5 min and cooled at 43°C±2°C. Subsequently, the sample was inoculated with 2 mL of the thermophilic lactic culture YC-180-40-FLEX (Chr. Hansen, Horshholm, Denmark; *Streptococcus thermophilus*, *Lactobacillus delbrűeckii* subsp. *lactis*, and *L. d*. subsp. *bulgaricus*). The inoculated sample was incubated at 43°C for 3.5 hours. The result was expressed as the titration acidity of the yogurt in mL of 0.25 mol×L^−1^ NaOH×100 mL^−1^ (or the so-called Soxhlet-Henkel degree) [25,26].

Rennetability (classical procedure) was determined during the tempering (35°C) of a defined milk volume after the addition of rennet (1% vol.) by measuring the time (rennet coagulation time RCT) until the first flakes of lactoproteins formed (beginning of coagulation). Rennetability was also determined by using nephelometry (turbidimetry measurement) to assess the milk coagulation time (ML – 2 analyzer). This is the use of the optical method (NEF, Nephelo - turbidimetric milk coagulation sensor ML – 2) to evaluate the intensity of the so-called diffusely scattered Tyndall light on dispersed particles (coagulating lactoprotein flakes) [26,27].

The milk ethanol stability, milk fermentation ability and milk rennetability are not introduced by an official standard as technological property in world literature references, but they are known according to citations in the scientific literature. These procedures were modified according to literature sources cited.

### Statistical analysis

Statistical analyses were performed using SAS (SAS 9.3, SAS Institute, Cary, NC, USA). Descriptive statistics for milk yield in kg, protein and fat percentages and protein and fat yield in kg during the first and second lactations are given in Supplementary Table S4. Descriptive statistics for the indicators of the technological quality of milk, i.e., the milk fermentation ability, rennetability assessed subjectively and instrumentally, and alcohol test are given in Supplementary Table S5. For the descriptive statistics and genotype frequencies in Supplementary Table S4, each record was assessed as a separate entry; when two lactations were recorded for the same cow, it was included twice. Similarly, for Supplementary Table S5, when a cow was measured repeatedly, the genotype was included repeatedly as well.

The data set contained repeated measurements per cow. Repeated measurements were obtained for the first and second lactation for milk performance traits. For technological quality, measurements were obtained several times over the course of two consecutive lactations. To analyze the influence of polymorphisms on milk yield and technological quality, the MIXED procedure of the SAS system with repeated measurements and the least squared mean method were used to compare genotypes. The models were developed as follows.

For milk performance traits, the following mathematical model was used:

$${\text{Y}}_{\text{ijk}}=\mu +{\text{gen}}_{\text{i}}+{\text{lac}}_{\text{j}}+{\text{anim}}_{\text{k}}+{\text{e}}_{\text{ijk}}$$

where Y_ijk_ = milk performance trait; μ = population mean; gen_i_ = fixed effect of the genotype (class effect i = 1, 2, 3); lac_j_ = fixed effect of the lactation order (class effect j = 1, 2); anim_k_ = random effect of the animal; and e_ijk_ = random residual effect.

Different mathematical models were used to determine the technological quality of milk:

$${\text{Yogurt}}_{\text{ijklmn}}=\mu +{\text{gen}}_{\text{i}}+{\text{farm}}_{\text{j}}+{\text{protein}}_{\text{k}}+{\text{casein}}_{\text{l}}+{\text{lacs}}_{\text{m}}+{\text{anim}}_{\text{n}}+{\text{e}}_{\text{ijklmn}}$$

where Yogurt_ijklmn_ = yogurt test values; μ = population mean; gen_i_ = fixed effect of genotype (class effect i = 1, 2, 3); farm_j_ = fixed effect of farm (class effect j = 1, 2, 3, 4, 5); protein_k_ = fixed effect of protein percentage content in milk; casein_l_ = fixed effect of casein content in milk; lacs_m_ = fixed effect of lactation stage in days; anim_n_ = random effect of the animal; and e_ijklmn_ = random residual effect.

$${\text{Rennetability}}_{\text{ijklmn}}=\mu +{\text{gen}}_{\text{i}}+{\text{farm}}_{\text{j}}+{\text{protein}}_{\text{k}}+{\text{NFS}}_{\text{l}}+{\text{season}}_{\text{m}}+{\text{anim}}_{\text{n}}+{\text{e}}_{\text{ijklmn}}$$

where Rennetability_ijklmn_ = rennetability assessed subjectively or instrumentally; μ = population mean; gen_i_ = fixed effect of genotype (class effect i = 1, 2, 3); farm_j_ = fixed effect of farm (class effect j = 1, 2, 3, 4, 5); protein_k_ = fixed effect of protein percentage content in milk; NFS_l_ = fixed effect of not fat solids content in milk; season_m_ = fixed effect of season (class effect m = 1, 2, 3, 4)*; anim_n_ = random effect of the animal; and e_ijklmn_ = random residual effect. * The fixed effect of season was created as a combination of three months according to natural weather conditions, temperature, pasture quality, etc. A year was divided into four seasons: 1 = December, January, February; 2 = March, April, May; 3 = June, July, August; and 4 = September, October, November.

$${\text{Ethanol}}_{\text{ijk}}=\mu +{\text{gen}}_{\text{i}}+{\text{farm}}_{\text{j}}+{\text{anim}}_{\text{k}}+{\text{e}}_{\text{ijk}}$$

where Ethanol_ijk_ = ethanol stability; μ = population mean; gen_i_ = fixed effect of genotype (class effect i = 1, 2, 3); farm_j_ = fixed effect of farm (class effect j = 1, 2, 3, 4, 5); anim_k_ = random effect of the animal; and e_ijk_ = random residual effect.

The effect of alleles on milk production traits and the technological quality of milk was computed using the following mathematical model:

$${\text{Y}}_{\text{ij}}=\mu +{\text{allele}}_{\text{i}}+{\text{anim}}_{\text{j}}+{\text{e}}_{\text{ij}}$$

where Y_ij_ = observed trait; μ = population mean; allele_i_ = fixed effect of allele (class effect i = 1,2); anim_j_ = random effect of the animal; and e_ij_ = random residual effect.

For post hoc comparisons, the Tukey-Kramer test was used [28].

## RESULTS AND DISCUSSION

### Milk yield and composition

In the *DGAT1* gene, the genotype AA and allele A, which codes for alanine, had a higher frequency than the genotype KA and the K allele, which codes for lysine (Supplementary Table S3); the homozygous genotype KK was not found at all. Other researchers found similarly unbalanced frequencies. In Israeli Holstein cows, the frequency of the K allele was reported to be 0.09 overall and 0.16 in sires [29]; in another study, the A allele had the highest frequency in dairy breeds, with the exception of Jersey [30]. Similarly, low frequencies of the K allele and KK homozygous genotype were found in Simmentals [31]. The A allele was confirmed repeatedly to be associated with higher milk, fat and protein yields, and its frequency in intensively selected populations increases due to indirect selection [14,32]. In our analysis, cows with the AA genotype outperform the heterozygous ones significantly in milk, protein and fat yields (Table 1). Additionally, when the effects of alleles are evaluated, A is advantageous but not significantly so (Table 2). This result is generally in agreement with previous findings and confirms our previous finding in German Holsteins regarding the trend of increasing frequency of the alanine variant [32,33].

Additionally, for the *LEP* gene, the MM genotype dominated (Supplementary Table S3). In Holstein cows, a reverse order of genotypes was also published [34]. MM homozygous cows had a lower protein percentage, and the difference between MM and MW was significant (Table 1). Allele W positively and significantly influenced the protein percentage (Table 2); differences in other indicators of milk performance were nonsignificant.

For the *FASN* gene, the protein content was slightly but significantly higher in GG homozygous cows. The A allele had significantly higher milk yield than the G allele, which resulted in significantly higher protein and fat yield. The differences in fat and protein percentages between alleles were negligible and nonsignificant (Table 2). The frequencies of allele G were markedly higher than those of A, which does not correspond fully with the differences between alleles in terms of performance. However, considering both genotypes and alleles, the performance differences were low, which may explain the differences in frequency.

The TT homozygous genotype in the *SCD1* gene was significantly associated with the lowest milk, protein and fat yields and with the highest protein contents (Table 1). The analysis of allele associations showed superiority of the T allele in all characteristics except fat content. The differences were not high but significant (Table 2). The differences among genotypes hint at intermediate heredity.

For the *CSN2* gene, the differences among genotypes were not significant. The B allele had significantly higher milk yield and therefore protein yield than A. In fat yield, the p-value was near the significance threshold. The differences in contents were low and nonsignificant (Tables 1, 2). Similarly, for the A^2^ and A^1^ genotypes, the effect was nonsignificant. Allele A^2^ was significantly better in terms of milk, protein and fat yields. The results of Ozdemir et al [35] indicated that none of the *CSN2* variants provide an advantage.

Genotype AA in the *CSN3* gene was significantly associated with high milk yield. BB homozygous cows had milk with a significantly higher protein percentage. Although the highest value was associated with the EE genotype, there were only two cows with a total of three lactations with this genotype, making it a minor consideration; similarly, for the BC genotype, there were two cows with four lactations. However, there were 21 cows of the BE genotype with 40 lactations, and they had significantly higher protein percentages comparing with the AA genotype (Table 1). The lowest protein content was found in AA homozygous cows, and the difference was significant. Thus, the positive influence of the B variant on the protein content was repeatedly shown. This was confirmed when evaluating the effect of alleles, specifically, the following significant effect was observed: E>B and B>A. However, the *CSN3* genotypes were not significantly associated with protein yield. Additionally, BB and B performed significantly better than AA and A in fat percentage. The differences in fat yield were not significant.

Our findings on the prevalence of the AA genotype and its effects on milk yield agree with other results found in Simmentals [36]. The authors also found the BB genotype to be associated with the highest protein percentage, but the fat percentage and yield were highest in milk from AA cows. In Czech Simmental cows, significant differences were reported among genotypes in daily milk yield, but the differences in protein and fat percentages were nonsignificant [37]. Ozdemir et al [35] conclude their review and meta-analysis by stating that the *CSN3* genotypes are ranked BB>AB>AA in terms of protein content and that the B allele could be considered a marker to improve milk protein content. The prevalence of the BB genotype in relation to protein yield was not always obvious. Additionally, for fat content, the BB genotype was better. They report that the associations of genotypes and alleles with milk yields were not significant. These findings are in general agreement with our results.

The AA genotype of the *CSN3* gene is usually the most frequent in both Black-and-White and Simmental cattle [20,36]. The frequencies of the BB genotype and B allele in our cows, both Holstein and Simmental, were rather low, which is also consistent with the frequencies found by other authors in Czech Simmental [6]. However, one other group of authors also found that, in Czech Simmental, the most frequent genotype was AB (0.487), and the frequency of the B allele was high (0.418) [37]. The frequency of the E allele (0.030) was the same as in our Simmental group (0.036). Apparently, there is leeway for breeding, and many Czech breeding companies report the genotype of the *CSN3* gene for the sires in their catalogues. However, changing genotype frequencies is a long-distance run.

For the *LGB* gene, the AB genotype was significantly associated with higher milk yield than in both homozygous genotypes, resulting in higher protein and fat yields. The rank of genotypes may indicate the effect of heterosis. Allele B outperformed A in milk, protein and fat yields, but the differences in the contents were not significant (Tables 1, 2). Additionally, other authors found significantly higher milk, protein yield and fat contents in Simmental cattle and higher fat yields (which were nonsignificant) in AB heterozygous cows [36].

The other factors potentially affecting milk performance were evaluated. Farm, breed and lactation order were tested in a general linear mixed model. The milk yield, fat yield and protein percentage were significantly influenced by all the factors. Fat content was influenced by farm, and protein yield was influenced by farm and lactation order. Thus, the importance of the effect of farms, i.e., specific stable, management, nutrition, veterinary care, milking, etc. was emphasized, even if some polymorphisms showed a significant association with milk performance.

### Milk technological characteristics

The testing of milk fermentation ability, renneting and ethanol stability was the final goal of our analysis. The topic is relevant because genetic background exerts a strong influence on the cheese-making properties of milk, largely due to genetic polymorphisms in the major milk protein genes [38]. In our analysis, the effect of alleles was not significant with the exception of *CSN2*, with the B allele outperforming the A allele (p<0.01) in terms of milk fermentation ability (yogurt test) (Table 2). The milk of KA heterozygous cows in the *DGAT1* gene significantly exceeded that of homozygous AA cows in milk fermentation ability (Table 3). The *LEP* gene had no significant effect, but the differences among genotypes in the alcohol test showed rising values in the order MM>MW>WW with significance differences of MM and MW vs WW at p<0.05. The differences among *FASN* genotypes were not significant. For the *SCD1* gene, the milk of TT cows was associated with significantly poorer performance in terms of renneting.

Certainly, the effects of polymorphous variants of milk protein genes are in focus. For the *CSN2* gene, the BB genotype had the best heat stability of milk as measured by the ethanol test (p<0.01), which is important for ultrahigh temperature milk production. However, with regard to renneting, the order was reversed. The A^1^A^1^ genotype was significantly associated with the best milk fermentation ability and was not significantly associated with renneting. Poulsen et al [39] refers to the negative association of A^2^ with coagulation. According to our results, it is difficult to describe the preferable genotype or allele in the *CSN2* gene.

Kappa-casein is the gene most often examined, as its influence on technological properties has been confirmed repeatedly. In our analysis, the rennetability measured instrumentally was significantly affected (p<0.05). Genotype BC was associated with the best milk, but only four measurements were performed; because of the high standard errors, this association is of limited importance. The BB genotype was associated with significantly better renneting than the AA genotype but not the AB genotype (Table 3). Genotypes with the A and E alleles (AE, AA, BE) were associated with the poorest rennetability. A probable explanation for the differences in milk protein coagulation is the changes in the primary amino acid sequence between the A and B variants of the kappa casein as a protective factor for raw milk casein micelles. The B variant of kappa casein differs from the A variant by amino acid substitutions at two positions: 136th, replacing threonine with isoleucine, and 148th, replacing asparagine with alanine. These changes in the amino acid sequence of the B variant may interact positively with the action of the rennet enzyme that starts cleavage of the casein molecule between the 105th and 106th amino acids of the peptide chain, i.e., not far from the changed amino acids.

For the milk fermentation ability, the p-value was close to the significance threshold. The ethanol test resistance was the best in milk from the cows with *CSN3* BB genotypes. The differences in these scores between the BB genotype on one hand and AA and AB genotypes on the other hand were significant at p<0.01. The difference BB>AE was significant at p<0.05. Overall, the advantage of the *CSN3* BB genotype was confirmed, but the B allele was not significantly better than the others (Table 2). Better properties for the BB genotype have also been reported by other authors [39,40], who stated a positive effect of *CSN2* B and *CSN3* B. They hinted at the additive genetic variation of milk coagulation and the possibility of selective breeding for variants associated with superior milk coagulation.

Lactoglobulin beta is the most important whey protein. In our analysis, the effect of genotype on ethanol number was significant at p<0.05, and BB was associated with a better value than AB at p<0.01.

When evaluating other factors, the farm was found to significantly affect all parameters of technological quality. Protein percentage was associated with the milk fermentation ability and rennetability scores, while fat percentage was associated with ethanol test performance. Nonfat solid content was associated with renneting, while somatic cell count was associated with renneting measured subjectively. The content of casein, month and season were associated with milk fermentation ability. The lactation order, day in milk and breed did not show significant effects. Thus, the key effect of the farm must be emphasized again. Interestingly, the farm with the best milk fermentation ability and rennetability had the worst ethanol test.

## CONCLUSION

According to our results, genotype AA and allele A of the *DGAT1* gene were associated with higher milk, protein and fat yields in kg, similar to allele A of the *FASN* gene, the B and A^2^ alleles of the *CSN2* gene, and the AB genotype and B allele of the *LGB* gene. Alleles B and E of the *CSN3* gene were associated with a higher protein percentage, allele B was also associated with the fat percentage, and genotype AA was associated with the milk yield. For the *SCD1* gene, allele T seemed to be associated with better scores in all characteristics of milk performance. Regarding the technological properties, the BB genotype of *CSN3* proved repeatedly to have merit, while the other genes did not show unequivocal influences in our field study. The importance of the effect of the farm on milk performance and milk quality was confirmed, so the effect of gene polymorphisms in the field conditions seemed to be somewhat blurred. Nevertheless, the polymorphisms in *DGAT1* and *CSN3* are ready for practical use in breeding. The polymorphisms in other genes should be a subject of further research.

## Figures and Tables

**Table 1:** Milk yield and composition according to genotype of Holstein and Czech Simmental cows

Gene	Genotype	n	Milk (kg)		Crude protein (%)		Protein (kg)		Fat (%)		Fat (kg)	
				
			LSM±SE	p-vale	LSM±SE	p-vale	LSM±SE	p-vale	LSM±SE	p-vale	LSM±SE	p-vale
*DGAT1*	AA	1,344	8,376^a^±84	0.045*	3.46±0.01	0.548	287.9^a^±2.6	0.027*	4.12±0.01	0.141	344.0^a^±3.4	0.019*
	KA	60	7,555^b^±401		3.49±0.04		260.1^b^±12.3		4.04±0.06		304.8^b^±16.3	
*LEP*	MM	925	8,412±101	0.156	3.45^a^±0.01	0.038*	287.7±3.1	0.281	4.11±0.01	0.909	344.7±4.1	0.235
	MW	229	8,138±203		3.50^b^±0.02		282.7±6.3		4.13±0.03		335.1±8.3	
	WW	45	7,667±450		3.51±0.04		266.4±13.9		4.11±0.06		317.5±18.4	
*FASN*	AG	378	8,527±158	0.180	3.43^a^±0.02	0.017*	290.5±4.9	0.371	4.13±0.02	0.562	349.8±6.4	0.177
	GG	1,018	8,277±97		3.48^b^±0.01		285.4±3.0		4.11±0.01		339.6±3.9	
*SCD1*	CC	398	8,549^A^±154	0.001**	3.43^A^^a^±0.01	<0.001**	290.5^A^±4.7	0.005**	4.10±0.02	0.606	348.4^A^±6.2	0.001**
	TC	811	8,426^A^±107		3.47^A^^b^±0.01		290.0^A^±3.3		4.12±0.02		346.9^A^±4.3	
	TT	187	7,608^B^±222		3.53^B^±0.02		266.4^B^±6.8		4.12±0.03		312.1^B^±9.0	
*CSN2*	AA	32	8,143±535	0.541	3.41±0.05	0.133	276.6±16.4	0.254	4.23±0.08	0.201	344.0±21.8	0.442
	AB	220	8,562±210		3.50±0.02		296.6±6.4		4.14±0.03		353.2±8.5	
	BB	1,105	8,326±93		3.46±0.01		286.0±2.8		4.11±0.01		341.2±3.8	
*CSN2*	A^1^A^1^	143	7,883±254	0.163	3.48±0.02	0.539	273.0±7.8	0.190	4.15±0.04	0.491	327.7±10.3	0.253
	A^1^A^2^	501	8,416±137		3.46±0.01		288.7±4.2		4.11±0.02		345.0±5.6	
	A^2^A^2^	675	8,217±120		3.48±0.01		283.0±3.7		4.10±0.02		335.7±4.9	
*CSN3*	AA	646	8,497^a^±96	0.112	3.43^A^,^X^±0.01	<0.001**	288.1±3.7	0.364	4.09^a^±0.02	0.133	345.4±4.9	0.377
	AB	586	8,267±101		3.50^B^,^Y^±0.01		287.0±3.9		4.13±0.02		341.1±5.2	
	BB	70	8,355±291		3.54^B^±0.03		292.3±11.5		4.20^b^±0.05		349.8±15.2	
	BC	4	8,140±1217		3.51±0.11		285.5±48.5		4.33±0.22		352.3±64.2	
	EE	3	5,627^b^±1406		3.79^B^,^Y^±0.13		199.2±50.8		4.24±0.23		221.8±67.1	
	AE	32	7,594^b^±430		3.47^Y^±0.04		258.0±16.2		4.20±0.06		311.8±21.4	
	BE	40	8,139±385		3.51^X^,^Y^±0.04		282.4±15.1		4.06±0.07		328.8±20.0	
*LGB*	AA	30	6,991^A^±546	<0.001**	3.52±0.05	0.611	245.9^A^±16.8	<0.001**	3.97±0.08	0.161	281.2^A^±22.2	<0.001**
	AB	1,222	8,471^B^±88		3.47±0.01		291.1^B^±2.7		4.12±0.01		347.9^B^±3.6	
	BB	103	7,515^A^±299		3.47±0.03		258.9^A^±9.2		4.11±0.04		309.1^A^±12.2	

n, number of lactations of cows with a particular genotype; LSM, least squared mean; SE, standard error; *DGAT1*, acyl-CoA diacylgycerol transferase 1; *LEP*, leptin; *FASN*, fatty acid synthase; *SCD1*, stearoyl CoA desaturase 1; *CSN2*, casein beta (*CSN2*); *CSN3*, casein kappa; *LGB*, lactoglobulin beta.

* Significant at p<0.05;

** significant at p<0.01.

a,b Different letters between genotypes in the same column represent significant differences at p<0.05.

A,B Different letters between genotypes in the same column represent significant differences at p<0.01.

X Differences between CSN3 genotypes AA and BE in the protein percentage are significant at p<0.05.

Y Differences between CSN3 genotypes EE on the one hand and AB, AE, BE on the other hand in the protein percentage are significant at p<0.05.

**Table 2:** Significance of differences in milk yield, composition, and qualities among alleles (p-values)

Gene		Milk (kg)	Crude protein (%)	Protein (kg)	Fat (%)	Fat (kg)	Milk fermentation ability (mL NaOH)	Renneting subjectively seconds	Renneting instrumentally seconds	Ethanol test mL of ethanol
*DGAT1*		0.861	0.255	0.753	0.308	0.628	0.064	0.528	0.354	0.659
*LEP*		0.999	0.023* W>M	0.701	0.835	0.904	0.446	0.823	0.642	0.077
*FASN*		0.008** A>G	0.896	0.009** A>G	0.610	0.007** A>G	0.142	0.906	0.555	0.536
*SCD1*		0.024* T>C	0.014* T>C	0.014* T>C	0.774	0.028* T>C	0.266	0.078	0.173	0.461
*CSN2* (A, B)		0.031* B>A	0.390	0.042* B>A	0.319	0.055	0.002** B>A	0.083	0.086	0.242
*CSN2* (A^1^,A^2^)		0.002** A^2^>A^1^	0.367	0.002** A^2^>A^1^	0.324	0.006**A^2^>A^1^	0.663	0.909	0.411	0.344
*CSN3*	A:B	0.187	<0.001** B>A	0.899	0.039* B>A	0.556	0.740	0.512	0.217	0.901
	A:E	0.046* A>E	0.314	0.055	0.111	0.099	0.817	0.240	0.914	0.526
	B:C	0.943	0.846	0.929	0.630	0.961	0.948	0.733	0.717	0.476
	B:E	0.068	0.010* E>B	0.074	0.815	0.062	0.091	0.980	0.322	0.440
*LGB*		<0.001** B>A	0.063	<0.001**B>A	0.736	<0.001** B>A	0.562	0.067	0.148	0.220

*DGAT1*, acyl-CoA diacylgycerol transferase 1; *LEP*, leptin; *FASN*, fatty acid synthase; *SCD1*, stearoyl CoA desaturase 1; *CSN2*, casein beta (*CSN2*); *CSN3*, casein kappa; *LGB*, lactoglobulin beta.

* Significant at p<0.05;

** significant at p<0.01.

**Table 3:** Milk technological qualities according to the genotype of Holstein and Czech Simmental cows

Gene	Genotype	Milk fermentation ability (mL NaOH)			Renneting assessed subjectively (seconds)			Renneting assessed instrumentally (seconds)			Ethanol test (mL of ethanol)		
			
		n	LSM±SE	p-vale	n	LSM±SE	p-vale	n	LSM±SE	p-vale	n	LSM±SE	p-vale
*DGAT1*	AA	435	14.93^A^±0.25	<0.001**	470	523.16±16.70	0.781	438	318.13±9.48	0.538	445	0.913±0.053	0.518
	KA	25	18.24^B^±0.87		31	507.79±53.79		23	338.64±32.64		25	1.052±0.204	
*LEP*	MM	288	15.09±0.33	0.709	315	510.28±18.78	0.609	289	314.68±10.54	0.654	293	0.864^a^±0.057	0.070
	MW	81	15.58±0.64		92	544.2±32.57		84	330.11±17.64		83	0.938^a^±0.105	
	WW	14	14.80±1.21		15	502.47±77.41		13	300.65±41.89		14	1.445^b^±0.248	
*FASN*	AG	118	15.07±0.44	0.998	130	545.46±26.47	0.262	117	336.70±14.53	0.123	115	1.026±0.094	0.202
	GG	338	15.07±0.28		367	512.17±18.52		340	311.87±10.37		351	0.888±0.058	
*SCD1*	CC	135	15.01±0.41	0.553	148	502.28^a^±25.10	0.029*	131	320.98±14.15	0.059	136	0.944±0.087	0.955
	TC	284	15.25±0.30		305	513.78^a^±19.74		287	309.75^a^±11.00		288	0.918±0.064	
	TT	41	14.54±0.67		48	625.41^b^±41.88		43	369.90^b^±24.21		46	0.898±0.151	
*CSN2*	AA	22	14.08±1.34	0.538	22	522.96±59.26	0.540	21	271.44^a^±30.86	0.121	22	0.448^A^±0.215	0.001**
	AB	171	15.39±0.45		187	498.54±26.77		172	304.21±14.68		176	0.724^A^±0.082	
	BB	267	14.98±0.32		292	532.28±19.03		268	329.06^b^±10.73		272	1.058^B^±0.062	
*CSN2*	A^1^A^1^	42	16.43^A^^a^±0.60	0.022*	45	527.46±48.38	0.968	38	314.22±26.72	0.462	43	0.829±0.153	0.769
	A^1^A^2^	148	14.60^B^±0.37		161	535.74±25.11		150	336.75±13.70		150	0.919±0.084	
	A^2^A^2^	224	15.17^b^±0.33		249	528.31±22.05		227	317.65±12.1		230	0.950±0.069	
*CSN3*	AA	215	15.22±0.34	0.075	228	552.38^a^±21.62	0.116	212	337.29^X^±12.02	0.037*	220	0.929^A^±0.070	0.109
	AB	191	15.04±0.37		216	504.38±22.32		196	299.82^X^±12.31		197	0.871^A^±0.073	
	BB	24	15.36±0.77		25	486.02±56.55		24	312.04±29.83		23	1.543^B^^a^±0.206	
	BC	4	14.77±1.35		4	299.68^b^±122.34		4	229.11^Y^±63.58		4	0.966±0.490	
	AE	13	14.97±1.00		15	454.31±68.79		13	331.77±39.04		13	0.701^b^±0.271	
	BE	12	12.89±1.93		12	616.24^a^±93.27		11	414.90^X^,^Y^±49.35		12	0.990±0.285	
*LGB*	AA	12	-	0.556	15	517.50±68.35	0.281	8	335.45±45.82	0.608	12	0.942±0.310	0.036*
	AB	390	15.15±0.27		416	510.01±18.42		393	314.56±10.45		394	0.857^A^±0.058	
	BB	58	14.76±0.64		70	576.97±37.91		60	337.37±21.73		64	1.216^B^±0.123	

n, number of samples from cows with a particular genotype; LSM, least squared mean; SE, standard error; *DGAT1*, acyl-CoA diacylgycerol transferase 1; *LEP*, leptin; *FASN*, fatty acid synthase; *SCD1*, stearoyl CoA desaturase 1; *CSN2*, casein beta (*CSN2*); *CSN3*, casein kappa; *LGB*, lactoglobulin beta.

* Significant at p<0.05;

** significant at p<0.01.

a,b Differences between genotypes with different letters in the same column are significant at p<0.05.

A,B Different letters between genotypes in the same column represent significant differences at p<0.01.

X Differences between CSN3 genotypes AB on the one hand and AA and BE on the other hand in renneting assessed instrumentally are significant at p<0.05.

Y Differences between CSN3 genotypes BE and BC in renneting assessed instrumentally are significant at p<0.05.
