# Supplementary material for: Gene polymorphisms influencing yield, composition and technological properties of milk from Czech Simmental and Holstein cows
Source: Anim Biosci. 2020 Jan 13;34(1):2–11. doi: 10.5713/ajas.19.0520 (PMC7888502; doi:10.5713/ajas.19.0520)
Supplement: Supplementary file 1 [file ajas-19-0520-suppl.pdf]

Table S1. The number of purebred and crossbred cows in the analysis

Breed	n	%
Holstein 100%	329	43.98
Holstein 75% + Simmental	17	2.27
Holstein 50% + Simmental, Ayrshire	12	1.60
Simmental 100%	259	34.63
Simmental 75% + Holstein	71	9.50
Simmental 75% + Ayrshire	40	5.35
Simmental 75% + Holstein, Ayrshire	20	2.67
Sum	748	100

Table S2. Sequences of primers used in the polymerase chain reactions

Locus	Primer	Sequence
DGAT1	Forward	5´ - GCA CCA TCC TCT TCC TCA AG – 3´
	Reverse	5´- GGA AGC GCT TTC GGA TG – 3´
LEPTIN	Forward	5´- ATG CGC TGT GGA CCC CTG TAT C – 3´
	Reverse	5´- TGG TGT CAT CCT GGA CCT TCC – 3´
FASN	Forward	5´- AGA GCT GAC GGA CTC CAC AC – 3´
	Reverse	5´- GCC GAT GCA CTC GAT GTA G – 3´
SCD1	Forward	5´- ACC TGG CTG GTG AAT AGT GCT – 3´
	Reverse	5´- TCT GGC ACG TAA CCT AAT ACC CT- 3´
CSN3	Forward	5´- TGT GCT GAG TAG GTA TCC TAG TTA TGG – 3´
	Reverse	5´- GCG TTG TCT TCT TTG ATG TCT CCT TAG – 3´
CSN2 (A^1, A^2)	Forward	5´- CCT TCT TTC CAG GAT GAA CTC CAG G – 3´
	Reverse	5´- GAG TAA GAG GAG GGA TGT TTT GTG GGA GGC TCT – 3´
CSN2 (A, B)	Forward	5´- CCA GAC ACA GTC TCT AGT CTA TCC C – 3´
	Reverse	5´- CAA CAT CAG TGA GAG TCA GGC TCC G – 3´
LGB	Forward	5´- TGT GCT GGA CAC CGA CTA CAA AAA G – 3´
	Reverse	5´- GCT CCC GGT ATA TGA CCA CCC TCT -3´

Table S3. Frequencies of genotypes and alleles of Holstein and Czech Simmental cows and crosses

		Cows of all breeds					Holstein cows and crosses				Czech Simmental cows and crosses			
Gene	Genotype	n	Relative frequencies	χ^2	Allele frequencies		n	Relative frequencies	χ^2	Allele frequencies	n	Relative frequencies	χ^2	Allele frequencies
DGAT1	*AA*	716	0.959	0.043	*A*	0.979	337	0.974	0.020	0.987	379	0.945	0.009	0.973
	KA	31	0.041		*K*	0.021	9	0.026		0.013	22	0.055		0.027
	KK	0	0.000				0	0.000			0	0.000		
LEP	*MM*	489	0.769	3.263	*M*	0.865	236	0.792	5.355	0.876	253	0.749	1.914	0.855
	MW	122	0.192		*W*	0.135	50	0.168		0.124	72	0.213		0.145
	WW	25	0.039				12	0.040			13	0.038		
FASN	*AA*	2	0.003	1.931	*A*	0.140	0	0	4.938	0.167	2	0.005	0.659	0.116
	AG	204	0.274		*G*	0.860	115	0.333		0.833	89	0.223		0.884
	GG	539	0.723				230	0.667			309	0.773		
SCD1	*CC*	207	0.279	3.681	*C*	0.571	113	0.328	6.663*	0.623	94	0.236	2.538	0.525
	CT	434	0.584		*T*	0.429	204	0.591		0.377	230	0.578		0.475
	TT	102	0.137				28	0.081			74	0.186		
CSN2	*AA*	18	0.025	2.257	*A*	0.105	12	0.037	7.748*	0.103	6	0.015	0.191	0.106

	AB	115	0.159		*B*	0.895	43	0.132		0.897	72	0.181		0.894
	BB	589	0.816				270	0.831			319	0.804		
CSN2	A^1A^1	80	0.114	0.887	A^1	0.306	39	0.125	0.313	0.335	41	0.105	1.553	0.283
	A^1A^2	270	0.385		A^2	0.694	132	0.422		0.665	138	0.355		0.717
	A^2A^2	352	0.501				142	0.454			210	0.540		
CSN3	*AA*	346	0.471	3.876	*A*	0.694	179	0.534	1.529	0.733	167	0.419	9.246	0.662
	AB	308	0.420		*B*	0.275	122	0.364		0.243	186	0.466		0.301
	BB	36	0.049		*C*	0.001	18	0.054		0.001	18	0.045		0.001
	AE	19	0.026		*E*	0.030	11	0.033		0.022	8	0.020		0.036
	BE	21	0.029				4	0.012			17	0.043		
	EE	2	0.003				0	0.000			2	0.005		
	BC	2	0.003				1	0.003			1	0.003		
LGB	*AA*	17	0.024	64.31^{**}	*A*	0.473	5	0.015	68.708^{**}	0.471	12	0.030	61.256^{**}	0.475
	AB	648	0.899		*B*	0.527	296	0.911		0.529	352	0.889		0.525
	BB	56	0.078				24	0.074			32	0.081		

*Significant differences between genotype frequencies calculated on the basis of Hardy-Weinberg equilibrium and empirical frequencies ($p < 0.05$)

**Significant differences between genotype frequencies calculated on the basis of Hardy-Weinberg equilibrium and empirical frequencies ($p < 0.01$)

Table S4. Descriptive statistics of the milk performance of Holstein and Czech Simmental cows

		Milk, kg		Protein, %		Protein, kg		Fat, %		Fat, kg	
Gene	Genotype	Mean	SD	Mean	SD	Mean	SD	Mean	SD	Mean	SD
DGAT1	*AA* n=1344	8392	2418	3.47	0.23	288.4	75.7	4.12	0.35	344.6	98.9
	KA n=60	7590	2607	3.49	0.26	261.3	79.7	4.04	0.30	306.4	102.8
LEP	*MM* n=925	8419	2429	3.45	0.23	287.9	75.6	4.11	0.35	345.0	98.4
	MW n=229	8112	2320	3.50	0.23	282.0	75.0	4.13	0.37	334.4	98.6
	WW n=45	7843	2938	3.50	0.25	271.4	96.1	4.13	0.40	325.1	121.8
FASN	*AG* n=378	8535	2475	3.44	0.22	290.8	77.3	4.13	0.36	350.2	101.0
	GG n=1018	8299	2417	3.48	0.23	286.0	75.6	4.11	0.34	340.5	98.8
SCD1	*CC* n=398	8587	2570	3.43	0.24	291.5	79.1	4.10	0.37	349.9	102.2
	TC n=811	8427	2401	3.47	0.22	290.2	76.1	4.13	0.34	347.0	99.6
	TT n=187	7627	2062	3.53	0.22	266.8	63.5	4.12	0.33	312.9	82.5
CSN2	*AA* n=32	8229	2663	3.41	0.29	279.3	85.9	4.25	0.35	349.1	114.1
	AB n=220	8602	2340	3.50	0.24	297.8	70.8	4.14	0.41	354.7	97.0
	BB n=1105	8334	2441	3.47	0.23	286.3	76.3	4.11	0.34	341.6	99.3
CSN2	A^1A^1 n=143	7931	2332	3.48	0.25	274.8	75.5	4.15	0.38	329.4	96.3
	A^1A^2 n=501	8418	2475	3.46	0.23	288.7	78.2	4.11	0.35	345.4	102.1
	A^2A^2 n=675	8240	2437	3.48	0.23	283.6	75.6	4.10	0.34	336.6	98.5
CSN3	*AA* n=646	8497	2431	3.43	0.23	288.5	74.9	4.09	0.34	345.9	96.6
	AB n=586	8267	2420	3.50	0.22	287.1	76.3	4.14	0.35	341.3	101.2
	BB n=70	8355	2658	3.54	0.23	293.9	88.2	4.22	0.37	352.1	109.7
	BC n=4	8140	405	3.51	0.12	285.5	19.5	4.33	0.22	352.3	27.3
	EE n=3	5627	2452	3.79	0.09	212.0	89.2	4.23	0.08	237.7	102.1
	AE n=32	7594	2535	3.47	0.20	261.8	84.0	4.20	0.43	317.1	108.1

	BE n=40	8139	2307	3.51	0.21	283.5	72.3	4.04	0.38	329.9	104.2
LGB	*AA* n=30	7066	2208	3.55	0.23	249.6	73.2	4.00	0.37	284.2	86.2
	AB n=1222	8484	2438	3.47	0.23	291.5	75.7	4.12	0.35	348.4	99.7
	BB n=103	7511	2115	3.47	0.21	258.6	68.2	4.12	0.35	309.2	85.5

n, number of lactations of cows with a particular genotype; SD, standard deviation

Table S5. Descriptive statistics of milk technological qualities of Holstein and Czech Simmental cows

Gene	Genotype	Milk fermentation ability, ml NaOH			Renneting assessed subjectively, seconds			Renneting assessed instrumentally, seconds			Ethanol test, ml of ethanol		
		n	Mean	SD	n	Mean	SD	n	Mean	SD	n	Mean	SD
DGAT1	*AA*	435	14.85	3.90	470	504.01	240.79	438	312.19	134.25	445	0.988	0.997
	KA	25	16.92	4.73	31	438.00	156.24	23	266.22	118.09	25	0.904	1.011
LEP	*MM*	288	15.38	4.03	315	495.02	216.87	289	310.95	136.11	293	0.902	0.831
	MW	81	14.66	3.90	92	503.55	273.26	84	308.83	141.95	83	0.992	1.058
	WW	14	15.34	4.15	15	478.80	351.77	13	264.92	122.52	14	1.436	1.746
FASN	*AG*	118	15.42	3.91	130	500.99	240.29	117	312.08	141.91	115	1.036	1.050
	GG	338	14.78	3.97	367	500.76	236.97	340	310.16	130.85	351	0.970	0.985
SCD1	*CC*	135	14.77	4.10	148	489.42	239.55	131	311.48	134.66	136	1.025	1.115
	TC	284	14.92	3.93	305	495.77	232.94	287	303.10	127.72	288	0.988	1.009
	TT	41	15.88	3.86	48	558.69	249.82	43	350.44	163.46	46	0.835	0.341
CSN2	*AA*	22	14.54	3.62	22	561.00	345.83	21	300.24	132.23	22	0.800	0.506
	AB	171	13.93	3.79	187	457.60	215.03	172	296.63	118.20	176	0.923	0.896
	BB	267	15.66	3.98	292	522.42	237.22	268	319.17	142.67	272	1.038	1.084
CSN2	A^1A^1	42	15.53	4.53	45	554.56	337.56	38	344.32	152.31	43	0.800	0.276
	A^1A^2	148	14.49	3.87	161	494.07	199.76	150	319.75	124.05	150	0.994	0.987
	A^2A^2	224	15.12	3.98	249	514.01	244.57	227	309.87	136.74	230	1.009	1.075
CSN3	*AA*	215	15.03	4.14	228	540.74	249.31	212	330.25	147.92	220	0.961	0.995
	AB	191	14.84	3.92	216	469.88	235.72	196	288.19	116.86	197	0.944	0.899
	BB	24	16.22	3.00	25	443.12	143.47	24	290.17	103.40	23	1.576	1.774
	BC	4	16.20	3.74	4	337.00	74.13	4	216.00	136.45	4	0.850	0.252

	EE	1	11.60	-	1	473.00	-	1	352.00	-	1	0.900	-
	AE	13	15.12	3.47	15	423.87	166.76	13	318.38	136.59	13	0.750	0.284
	BE	12	12.88	3.83	12	535.25	145.28	11	367.64	126.23	12	1.217	0.980
LGB	*AA*	12	15.71	4.79	15	426.60	184.67	8	233.88	80.23	12	0.650	0.294
	AB	390	14.89	3.95	416	492.02	232.22	393	307.16	134.32	394	0.950	0.960
	BB	58	15.28	4.03	70	562.60	264.13	60	338.00	131.15	64	1.252	1.239

n, number of samples from cows with a particular genotype; SD, standard deviation
